# Clinical, Functional and Health-Related Quality of Life Correlates of Clinically Significant Symptoms of Anxiety and Depression in Patients with Systemic Sclerosis: A Cross-Sectional Survey

**DOI:** 10.1371/journal.pone.0090484

**Published:** 2014-02-28

**Authors:** Christelle Nguyen, Brigitte Ranque, Thierry Baubet, Alice Bérezné, Caroline Mestre-Stanislas, François Rannou, Agathe Papelard, Sandrine Morell-Dubois, Michel Revel, Marie-Rose Moro, Loïc Guillevin, Serge Poiraudeau, Luc Mouthon, on behalf of the Groupe Français de Recherche sur la Sclérodermie

**Affiliations:** 1 Pôle de Médecine Interne, Centre de Référence pour les Vascularites Nécrosantes et la Sclérodermie Systémique, Hôpital Cochin, Faculté de Médecine Paris Descartes, Université Paris Descartes & Assistance Publique-Hôpitaux de Paris, Paris, France; 2 Service de Médecine Interne, Hôpital Européen Georges Pompidou, Faculté de Médecine Paris Descartes, Université Paris Descartes & Assistance Publique-Hôpitaux de Paris, Paris, France; 3 Service de Psychopathologie, Hôpital d’Avicenne, EA 3413, Université Paris XIII & Assistance Publique-Hôpitaux de Paris, Bobigny, France; 4 Service de Médecine Physique et Réadaptation, Hôpital Cochin, Faculté de Médecine Paris Descartes, Université Paris Descartes & Assistance Publique-Hôpitaux de Paris, INSERM, Institut Fédératif de Recherche sur le Handicap, Paris, France; 5 Service de Médecine Interne, Centre de Référence pour la Sclérodermie Systémique, Hôpital Claude Huriez, Université Lille 2, Lille, France; 6 Service de Psychopathologie de l’Adolescent, Hôpital Cochin, Faculté de Médecine Paris Descartes, Université Paris Descartes & Assistance Publique-Hôpitaux de Paris, INSERM U669, Paris, France; Keio University School of Medicine, Japan

## Abstract

**Objectives:**

To identify clinical, functional and health-related quality of life (HRQoL) correlates of clinically significant symptoms of anxiety and depression in patients with systemic sclerosis (SSc).

**Methods:**

Three-hundred-and-eighty-one patients fulfilling the American College of Rheumatology and/or the Leroy and Medsger criteria for SSc were assessed for visceral involvement, disability and HRQoL (assessed by SF-36). Clinically significant symptoms of anxiety and depression were evaluated with the Hospital Anxiety Depression Scale (HAD) (defined cut-off≥8).

**Results:**

9.2% the patients had limited SSc, 50.5% limited cutaneous SSc (lcSSc), and 40.3% diffuse cutaneous SSc (dcSSc). Overall, 40.4% and 58.8% of the patients had clinically significant symptoms of depression and anxiety, respectively. Compared to patients without clinically significant symptoms of depression, patients with clinically significant symptoms of depression had poorer health status, HRQoL mental and physical component, and greater global disability, hand disability and aesthetic impairment. Compared to patients without clinically significant symptoms of anxiety, patients with clinically significant symptoms of anxiety had poorer SF-36 mental and physical component scores. On multivariable analysis, excluding mental component score of SF-36, variables independently associated with clinically significant symptoms of depression and anxiety were global disability and physical component of SF-36, plus female gender for clinically significant symptoms of anxiety only. Remarkably, patients with and without clinically significant psychiatric symptoms were comparable for all disease-related clinical features assessed.

**Conclusion:**

High levels of clinically significant symptoms of anxiety and depression are observed among SSc patients. Clinically significant psychiatric symptoms are rather associated with increased disability and altered HRQoL, than with disease-specific organ manifestations.

## Introduction

Systemic sclerosis (SSc) is a connective-tissue disease characterized by excessive collagen deposition in the dermis and internal organs and by vascular hyper-reactivity and obliterative microvascular phenomena [1]. SSc is classified according to the extent of skin involvement. Limited SSc (lSSc) features no detectable skin involvement. Limited cutaneous SSc (lcSSc) is characterized by skin sclerosis limited to the hands and face, with relatively rare visceral involvement [2], [3]. Diffuse cutaneous SSc (dcSSc) features proximal skin involvement to the elbows and knees and frequent visceral involvement, and is associated with decreased survival [4]. In addition to diminishing life expectancy, SSc is responsible for skin, tendon, joint, and vessel damage, which ultimately leads to disability and compromised health-related quality of life (HRQoL) [5]. Patients with SSc also frequently experience clinically significant psychiatric symptoms [6].

The prevalence of clinically significant symptoms of depression in SSc varies between 36% and 65% as a consequence of the method of detection/evaluation used [7]–[18]. Depression symptoms probably result from disease chronicity, reduced life expectancy, and disability [7]–[18]. Several predictors of clinically significant symptoms of depression have been inconsistently identified in this condition, including education level, gastrointestinal tract involvement, pain, or psychological constructs. Although some studies found that SSc severity was a predictor of clinically significant symptoms of depression, other studies did not find links with indices of disease severity or disease duration [19]. This discrepancy may be explained by published studies’ limitations, including small sample sizes, absence of a control group, relatively weak methodological approaches and recruitment from a single centre [19].

Surprisingly, only few studies have assessed clinically significant symptoms of anxiety in SSc patients [7], [12], [17], [20], [21]. In a recent Serbian study comparing 35 patients with SSc to 30 age- and gender-matched healthy individuals, clinically significant symptoms of anxiety were found in 80% of patients with SSc compared with 13% of healthy individuals [21]. In this study, no significant association was found between disease severity or socioeconomic factors and the development of clinically significant symptoms of anxiety [21]. Recently, we found more frequent self-reported clinically significant symptoms of anxiety in SSc females [22]. However, the above-mentioned studies were performed with various dimensional scales and very small sample size, so that only little consistency was found.

Because of the few reports and their limitations, no clear clinical correlates of clinically significant symptoms of depression and/or anxiety emerge in SSc patients. In the present study, we aimed to identify clinical manifestations, and disease-related handicap and HRQoL features associated with clinically significant symptoms of depression and/or anxiety, in a cohort of French patients with SSc, using established cut-offs with the Hospital Anxiety and Depression Scale (HAD).

## Patients and Methods

### Study Design

We performed a cross-sectional survey of 381 patients. Patients with SSc were prospectively included during 7 consecutive annual meetings of the French SSc patient association, the “Association des Sclérodermiques de France” (ASF), between 2003 and 2009, or during their hospitalization in Cochin (between January 2006 and June 2009) or Claude Huriez (between January and June 2009) hospitals. Since some patients were evaluated during several ASF annual meetings, only the most recent assessment of each patient was considered. Patients had to complete self-administered questionnaires first and then to undergo an interview with a physician to check for unanswered questions, fully complete questionnaires, and gather clinical data.

### Patients

To be eligible for the study, patients had to fulfill the American College of Rheumatology [23] and/or the Leroy and Medsger [24] criteria for SSc. Patients from the ASF were assessed within 48 hrs. during spring (temperature around 20°C).

### Measures

Parameters recorded were age; sex; age at disease onset; disease duration; body mass index (BMI); disease subset (lSSc, lcSSc or dcSSc); Karnofsky Performance Status (KPS) score; mouth opening (inter-incisor distance measured in millimetres); skin involvement; telangiectasia; Raynaud’s phenomenon; pitting scars; digital ulcers; calcinosis; gastrointestinal tract, joint and/or muscle involvement; dyspnoea (assessed by the New York Heart Association [NYHA] 4-point scale); interstitial lung disease (ILD); echocardiography systolic pulmonary artery pressure (PAP) >35 mmHg; and renal crisis. History of esophagus, gastrointestinal, joint, muscle and/or heart involvement; ILD; and renal crisis was obtained from detailed clinical charts for hospitalized patients and self-reports for ASF members.

### Health Status

Health status was assessed by the KPS score, the scale ranging from 0 (dead) to 100 (normal no complaints; no evidence of disease) [25]. This scale has already been used in SSc [22], [26]–[32], but not specifically validated.

### Health-related Quality of Life

HRQoL was assessed by the French version of the Medical Outcomes Study 36-Item Short Form Health Survey (SF-36) [33], a self-administered questionnaire covering 8 areas: physical function, physical role, bodily pain, general health, vitality, social function, emotional role, and mental health. For each area, scores range from 0 (poorer health status) to 100 (better health status). Scores can also be summarized in 2 global scores: physical component score (PCS) and mental component score (MCS). This scale has already been used in SSc [22], [26]–[32]. Published data suggest a good responsiveness in SSc [32].

### Disability

#### Global disability

Global disability was assessed by the Health Assessment Questionnaire (HAQ), the score ranging from 0 (no disability) to 3 (maximal disability). The HAQ includes 20 items divided into 8 domains: dressing/grooming, arising, eating, walking, hygiene, reach, grip, common daily activities [34]. This scale has already been used in SSc [22], [26]–[32]. It has demonstrated reliability [35] and construct, concurrent, and predictive validities in SSc [36].

#### Patients’ perceived disability

Patients’ perceived disability was assessed by the McMaster Toronto Arthritis Patient Preference Disability Questionnaire (MACTAR) [37]. Patients were asked to select the 3 situations among activities of daily living (ADL) that caused them maximal trouble. Each item is scored on an 11-point semi-quantitative scale (range 0–10). The global score ranges from 0 (no disability) to 30 (maximal disability) [29]. Construct validity and sensitivity to change of this score have been addressed in SSc [29], [30].

#### Hand disability

Hand disability was assessed by the Cochin Hand Function Scale (CHFS) [38], a questionnaire administered by the physician that contains 18 items related to ADL. Each question is scored on a scale of 0 (performed without difficulty) to 5 (impossible to do). The total score is obtained by adding the scores of all items (range 0–90). This questionnaire has shown good reproducibility, as well as satisfying construct and concurrent validities in SSc [32], [39]. Recent review of its psychometric properties supports its use in clinical trials [40].

#### Mouth disability

Mouth disability was assessed by the Mouth Handicap In Systemic Sclerosis (MHISS) scale, a questionnaire administered by the physician that contains 12 items concerning difficulties in performing ADL. Each question is scored on a scale of 0 (never) to 4 (always) [28]. The total score is obtained by adding the scores of all items (range 0–48). The French, Italian and Dutch versions of this scale have shown reliability and good construct validity in SSc [28], [41], [42].

### Clinically Significant Symptoms of Anxiety and Depression

Clinically significant symptoms of anxiety and depression were assessed by the HAD. This scale has 7 questions for the anxiety dimension (HADa) and 7 for the depression dimension (HADd). Each item is scored on a scale of 0 to 3, the total score ranging from 0 (no clinically significant symptoms of depression or of anxiety) to 21 (maximal clinically significant symptoms of depression or of anxiety). Scores of 0–7 in subscales are considered normal, 8–10 borderline and ≥11 clinical caseness [43]. The definition of clinically significant symptoms of anxiety and/or depression was based on the HAD score cut-off ≥8 found to be relevant in patients with autoimmune diseases and SSc [15], [44]. This scale has already been used in SSc [22], [26]–[32], but not specifically validated.

### Aesthetic Impairment

Aesthetic impairment was assessed on an 11-point semi-quantitative scale, the total score ranging from 0 (no aesthetic impairment) to 10 (maximal aesthetic impairment).

### Statistical Analysis

Data analysis involved use of Systat 9 software (SPSS Inc., Chicago, IL, USA). Quantitative variables were described with median [inter-quartile range (IQR)] and qualitative variables with counts and percentages. To identify parameters associated with clinically significant symptoms of depression and anxiety, demographic, clinical and functional characteristics were compared between patients with or without clinically significant symptoms of depression and between patients with or without clinically significant symptoms of anxiety, respectively. For bivariable analysis, comparisons involved Pearson Chi-Square test for qualitative variables and Wilcoxon test for quantitative data. Bonferroni adjustment was used for multiple comparisons (40 comparisons); therefore a *p* value less than 0.001 was considered statistically significant. Multivariable analysis was then conducted to determine the variables independently associated with patients’ clinically significant symptoms of anxiety and depression. Two separate hierarchical multivariable logistic regressions were performed, one with clinically significant symptoms of anxiety as the outcome, and the other with clinically significant symptoms of depression as the outcome, for demographic, clinical variables, and HRQoL- and function-related variables, with adjustment for age, sex, disease duration and recruitment type. On step 1, we included demographic variables. On step 2, we added disease-related clinical variables that were previously reported associated with clinically significant psychiatric disorders, including gastrointestinal tract involvement and dyspnoea. Other clinical disease-related variables related to pain (myalgia and arthralgia) or that yielded a *p* value less than 0.15 on bivariable analysis were also included. On step 3, we added SF-36 physical component score and function-related variables that yielded a *p* value less than 0.15 on bivariable analysis. SF-36 mental component scores were not included in the model because of obvious strong association with both clinically significant symptoms of depression and anxiety that could mask other associations. MHISS and CHFS were not included because of missing data for more than half of patients. Entered variables were selected using backward stepwise regression with values of 0.15 to enter in the model and of 0.15 to stay. A *p* value less than 0.05 was considered significant in the final multivariable model.

### Ethical Considerations

This survey was conducted in compliance with the protocol of Good Clinical Practices and Declaration of Helsinki principles. Patients gave their consent to participate after being orally informed about the study protocol. In accordance with European regulation (Directive 2001/20/EC of the European Parliament and of the Council of 4 April 2001 on the approximation of the laws, regulations and administrative provisions of the Member States relating to the implementation of good clinical practice in the conduct of clinical trials on medicinal products for human use; Directive 95/46/EC of the European Parliament and of the Council of 24 October 1995 on the protection of individuals with regard to the processing of personal data and on the free movement of such data), French observational studies from data obtained without any additional therapy or monitoring procedure, do not need formal approval of an Institutional Review Board or an Independent Ethics Committee, and a formal written consent from the patients is not required for this kind of project.

## Results

### Demographic and Clinical Data

Overall, 381 patients were included: 143 of them were recruited during their hospitalization in Cochin (n = 135) or Claude Huriez (n = 16) hospitals, and the remaining 238 were recruited during ASF annual meetings from 2003 to 2009. The proportion of patients from the ASF who agreed among those who were asked to participate were 51 among 80 (63.8%) (44 females) in 2003, 50 among 80 (62.5%) (44 females) in 2004, 71 among 98 (72.4%) (59 females) in 2005, 70 among 95 (73.7%) (55 females) in 2006, 70 among 101 (69.3%) (55 females) in 2007, 86 among 130 (66.1%) (74 females) in 2008 and 2009 altogether. Of the 381 patients, 62 were males (16.4%), with a female to male ratio of 5∶1. The median age at the time of evaluation was 57 (47–65) years, and median disease duration was 7 (3–13) years. A total of 149 (40.3%) patients had dcSSc, 187 (50.5%) had lcSSc, and 34 (9.2%) had lSSc. The median KPS was 80 (70–90) (Table 1).

**Table 1:** Demographic and clinical characteristics of 381 patients with systemic sclerosis*.

Age, years, median (IQR)	57 (47–65)
Age at disease onset, years, median (IQR)	46 (38–55)
Male sex	62/379 (16.4)
Patient association	62/191 (32.5)
Disease duration, years, median (IQR)	7 (3–13)
Body mass index, kg/m^2^, median (IQR)	23 (20–26)
Diffuse cutaneous SSc	149/370 (40.3)
Limited cutaneous SSc	187/370 (50.5)
Limited SSc	34/370 (9.2)
KPS (0–100), median (IQR)	80 (70–90)
Inter-incisor distance, mm, median (IQR)	36 (30–40)
Skin involvement	339/370 (91.6)
Telangiectasia	253/347 (72.9)
Raynaud’s phenomenon	369/377 (97.9)
Pitting scars	221/376 (58.8)
Digital ulcers	170/375 (45.3)
Calcinosis	105/312 (33.7)
Gastrointestinal tract involvement	304/375 (81.1)
Arthralgia	254/375 (67.7)
Myalgia	209/375 (55.7)
Dyspnea, NYHA classification, median (IQR)	2 (2–3)
Interstitial lung disease	163/373 (43.7)
Echocardiography systolic PAP>35 mmHg	48/375 (12.8)
Scleroderma renal crisis	34/375 (9.1)

*Values are number/number of patients for whom the data is available (%), otherwise indicated.

IQR: interquartile range; KPS: Karnofsky performance status; NYHA: New York Heart Association; PAP: pulmonary artery pressure.

### Levels of Clinically Significant Symptoms of Anxiety and Depression in Patients with SSc

Overall, clinically significant symptoms of depression were detected in 154 patients (40.4%) and anxiety in 224 patients (58.8%). Two-hundred-and-fifty patients (66.1%) displayed clinically significant symptoms of anxiety and/or of depression, whereas 129 (33.9%) were free of both clinically significant symptoms of anxiety and of depression. Ninety-six patients (25.2%) had clinically significant symptoms of anxiety only, whereas 26 (6.8%) had clinically significant symptoms of depression only (Table 2). We observed no significant difference of clinically significant symptoms of depression and anxiety prevalences according to the type of recruitment: clinically significant symptoms of depression prevalence was 63/129 (50.4%) *vs* 25/62 (40.3%) p = 0.269; and clinically significant symptoms of anxiety prevalence was 81/129 (62.8%) *vs* 36/62 (58.1%), p = 0.530), for hospitalized and ASF patients, respectively.

**Table 2:** Clinically significant symptoms of depression and anxiety in 381 patients with systemic sclerosis*.

HADa, median (IQR)	9 (6–12)
HADd, median (IQR)	6 (3–9)
HADs, median (IQR)	15 (10–21)
Free of psychiatric symptoms (HADa<8 and HADd<8)	129 (33.9)
Anxiety symptoms (HADa≥8)	224 (58.8)
Depression symptoms (HADd≥8)	154 (40.4)
Both anxiety and depression symptoms (HADa≥8 and HADd≥8)	128 (33.6)
Anxiety symptoms only (HADa≥8 and HADd<8)	96 (25.2)
Depression symptoms only (HADd≥8 and HADa<8)	26 (6.8)

*Values are number (%), otherwise indicated.

HADa: Hospital Anxiety and Depression scale for Anxiety; HADd: Hospital Anxiety and Depression scale for Depression; HADs: Hospital Anxiety and Depression scale.

### Clinical Features Associated with Clinically Significant Symptoms of Anxiety and Depression in Patients with SSc

Patients with clinically significant symptoms of anxiety were comparable to patients without clinically significant symptoms of anxiety for all the clinical parameters assessed. Patients with clinically significant symptoms of depression were similar to patients without clinically significant symptoms of depression for every clinical parameters studied, except for health status as assessed by the KPS score (Table 3).

**Table 3:** Clinical features associated with clinically significant symptoms of anxiety and depression in patients with systemic sclerosis*.

	Patients with anxiety symptoms	Patients without anxiety symptoms	p-value	Patients with depression symptoms	Patients without depression symptoms	p-value
	n = 224	n = 157		n = 154	n = 227	
Age, years, median (IQR)	57 (46–66)	57 (47–65)	0.844	58 (47–66)	55 (48–65)	0.229
Age at disease onset, years, median (IQR)	46 (39–55)	47 (37–54)	0.993	46 (39–55)	46.5 (37–53)	0.210
Male sex	27/224 (12.1)	35/155 (22.6)	0.006	25/154 (16.2)	37/225 (16.4)	0.957
Patient association	36/117 (30.8)	26/74 (35.1)	0.530	25/88 (28.4)	37/103 (35.9)	0.269
Disease duration, years, median (IQR)	7 (3–14)	7 (4–13)	0.435	8 (4–14)	7 (3–13)	0.711
Body mass index, kg/m^2^, media (IQR)	23 (20–27)	23 (20–26)	0.528	23 (20–26)	23 (21–26)	0.728
Diffuse cutaneous SSc	81/218 (37.2)	68/151 (45.0)	0.129	64/148 (43.2)	85/221 (38.5)	0.359
Limited cutaneous SSc	116/218 (53.2)	70/150 (4.7)	0.217	76/148 (51.4)	110/220 (50.0)	0.799
Limited SSc	21/218 (9.6)	13/151 (8.6)	0.738	8/148 (5.4)	26/221 (11.8)	0.038
KPS (0–100), median (IQR)	80 (70–80)	80 (70–90)	0.071	80 (70–80)	80 (70–90)	<0.001*
Inter-incisor distance, mm, median (IQR)	38 (30–42)	35 (30–40)	0.093	39 (30–42)	35 (28–40)	0.005
Skin involvement	199/219 (90.9)	138/151 (91.4)	0.862	143/151 (94.7)	194/219 (88.6)	0.042
Telangiectasia	149/207 (72.0)	102/140 (72.9)	0.858	101/141 (71.6)	150/206 (72.8)	0.809
Raynaud’s phenomenon	219/223 (98.2)	148/154 (96.1)	0.212	150/153 (980.)	217/224 (96.9)	0.490
Pitting scars	130/224 (58.0)	90/152 (59.2)	0.821	95/153 (62.1)	125/223 (56.1)	0.243
Digital ulcers	103/223 (46.2)	67/152 (44.1)	0.687	78/152 (51.3)	92/223 (41.3)	0.055
Calcinosis	61/185 (33.0)	42/127 (33.1)	0.986	43/122 (35.2)	60/190 (31.6)	0.502
Gastrointestinal tract involvement	184/223 (82.5)	119/152 (78.3)	0.308	130/153 (85.0)	173/222 (77.9)	0.089
Arthralgia	156/224 (69.6)	96/151 (45.7)	0.220	116/153 (75.8)	136/222 (61.3)	0.003
Myalgia	135/223 (60.5)	73/152 (48.0)	0.017	95/153 (62.1)	113/222 (51.0)	0.032
Dyspnea, NYHA classification, median (IQR)	2 (2–3)	2 (2–3)	0.696	2 (1–3)	2 (2–3)	0.003
Interstitial lung disease	90/220 (41.0)	73/153 (47.7)	0.193	66/150 (44.0)	97/223 (43.5)	0.924
Echocardiography systolic PAP>35 mmHg	31/223 (13.9)	17/152 (11.2)	0.439	26/152 (17.1)	22/223 (9.9)	0.039
Scleroderma renal crisis	27/223 (12.1)	7/152 (4.6)	0.013	18/153 (11.8)	16/222 (7.2)	0.131

Values are number/number of patients for whom the data is available (%), otherwise indicated.

*After Bonferroni correction for multiple comparisons, a p-value less than 0.001 was considered statistically significant.

IQR: interquartile range; KPS: Karnofsky Performance Status; n: number; NYHA: New York Heart Association; PAP: pulmonary artery pressure; SSc: systemic sclerosis.

### Functional Outcomes Associated with Clinically Significant Symptoms of Anxiety and Depression in Patients with SSc

Hand disability and mouth handicap were significantly higher in patients with clinically significant symptoms of anxiety. Likewise, compared to patients without clinically significant symptoms of depression, patients with clinically significant symptoms of depression had more severe global disability, hand disability, mouth handicap and aesthetic impairment (Table 4).

**Table 4:** Disability and health-related quality of life features associated with clinically significant symptoms of anxiety and depression in patients with systemic sclerosis.

	All patients n = 381	Patients with anxiety symptoms	Patients without anxiety symptoms	p-value	Patients with depression symptoms	Patients without depression symptoms	p-value
		n = 224	n = 157		n = 154	n = 227	
SF-36							
• Physical functioning	26 (18–50)	25 (15–50)	26 (20–55)	0.011	25 (16–50)	27(21–55)	0.015
• Physical role	5 (4–8)	4 (0–8)	6 (4–25)	0.003	4 (4–8)	7 (4–25)	<0.001*
• Bodily pain	5 (3–41)	2 (5–34)	5 (3.41)	<0.001*	5 (3–41)	6 (4–41)	0.053
• General health perception	12 (9–35)	13 (9–30)	11 (9–44)	0.005	11 (9–30)	12 (9–49)	0.773
• Vitality	10 (6–35)	8 (5–31)	10 (6–40)	<0.001*	8 (7–35)	11 (7–45)	0.063
• Social functioning	5 (3–50)	5 (3–63)	5 (3–50)	<0.001*	4 (3–50)	5 (4–75)	0.004
• Emotional role	5 (3–33)	3 (0–5)	6 (3–67)	<0.001*	3 (2–6)	6 (5–100)	<0.001*
• Mental health	17 (13–56)	15 (10–44)	18 (14–60)	<0.001*	15 (11–44)	18 (15–65)	<0.001*
• PCS	35 (29–42)	33 (27–39)	36 (29–44)	<0.001*	33 (28–39)	38 (30–45)	0.002
• MCS	34 (25–41)	28 (21–34)	37 (29–38)	<0.001*	29 (23–37)	39 (33–49)	<0.001*
HAQ (0–3)	1 (0.5–1.6)	1.1 (0.5–2)	0.9 (0.4–1.5)	0.009	1.1 (0.5–1.8)	0.8 (0.4–1.5)	<0.001*
MACTAR (0–30)	19.5 (15–24)	18.5 (13–24)	18.5 (15–25)	0.192	20 (15–24)	18 (13–24)	<0.001*
CHFS (0–90)	14 (4–30)	20 (7–42)	12 (3–27)	<0.001*	19 (6–35)	10 (2–25)	<0.001*
MHISS (0–48)	19.5 (9–27)	18 (18–28)	15 (6–24.5)	<0.001*	23 (15–27)	11 (4–25)	<0.001*
Aesthetic impairment (0–10)	5 (3–7)	5 (4–7)	4 (2–6)	0.068	5 (4–7)	4 (2–6)	<0.001*

Values are given in median (interquartile range).

*After Bonferroni correction for multiple comparisons, a p-value less than 0.001 was considered statistically significant.

CHFS: Cochin Hand Function Scale; HAQ: Health Assessment Questionnaire; MACTAR: McMaster-Toronto Arthritis Patient Preference Disability Questionnaire; MCS: Mental Component Score; MHISS: Mouth Handicap In Systemic Sclerosis Scale; n: number; PCS: Physical Component Score; SF-36: Medical Outcomes Study 36-Item Short Form Health Survey.

### Health-related Quality of Life Features Associated with Clinically Significant Symptoms of Anxiety and Depression in Patients with SSc

Compared to patients without clinically significant symptoms of anxiety, patients with clinically significant symptoms of anxiety had poorer HRQoL as given by significantly lower MCS and PCS in bivariable analysis. Likewise, compared to patients without clinically significant symptoms of depression, patients with clinically significant symptoms of depression exhibited altered HRQoL as summarized by significantly lower MCS and PCS in bivariable and in multivariable analysis (Table 4).

### Clinical, Functional and Health-related Quality of Life Correlates of Clinically Significant Symptoms of Anxiety and Depression in Patients with Systemic Sclerosis

On multivariable analysis, clinically significant symptoms of depression were independently associated with global disability assessed by the HAQ and poorer HRQoL PCS. Variables independently associated with clinically significant symptoms of anxiety were female sex, HAQ (positively) and PCS (negatively). Adjusted odds ratios and 95% confidence intervals of the final model’s variables are summarized in Table 5.

**Table 5:** Final model of multivariable analysis of current clinically significant symptoms of depression and anxiety according to clinical, functional and health-related quality of life features of patients with systemic sclerosis, with adjustment for age and sex.

Variables	AdjustedOdds Ratio	95% CI	P value
Depression symptoms correlates			
• Age	1.01	0.99–1.03	0.354
• Sex	1.17	0.55–1.86	0.768
• HAQ	2.90	0.74–1.38	<10^9^
• SF36 -PCS			0.005
Anxiety symptoms correlates			
• Age	1.00	0.98–1.01	0.810
• Sex	0.49	0.57–1.75	0.003
• HAQ	1.40	0.76–1.31	0.012
• SF36 -PCS			0.002

A p-value less than 0.05 was considered statistically significant.

CI: confidence interval; HAQ: Health Assessment Questionnaire; PCS: Physical Component Score;; of SF-36: Medical Outcomes Study 36-Item Short Form Health Survey.

## Discussion

In the present study, 58.8% and 40.4% of patients had current clinically significant symptoms of anxiety or depression, respectively, whereas 33.9% were free of both clinically significant symptoms of anxiety and of depression, using the easy to use self-rated psychiatric questionnaire HAD with a cut-off of 8 [15], [44]. Compared to patients without clinically significant symptoms of anxiety, patients with clinically significant symptoms of anxiety had poorer HRQol and greater mouth and hand disabilities. Variables independently associated with clinically significant symptoms of anxiety were female sex, HAQ and SF-36 PCS. Compared to patients without clinically significant symptoms of depression, patients with clinically significant symptoms of depression had poorer health status and HRQoL PCS, and greater global and localized disabilities and aesthetic impairment. Variables independently associated with clinically significant symptoms of depression were HAQ and SF-36 PCS. Remarkably, patients with and without clinically significant psychiatric symptoms were comparable for all disease-related clinical features assessed.

In our study, we found high levels of both current clinically significant symptoms of depression and anxiety using the HAD. In a previous study of 100 French SSc patients using the Mini International Neuropsychiatric structured Interview (MINI), the prevalence of current and lifetime major depressive episode was high and reached 19% and 56% respectively, while 14% of the patients had current dysthymia [45]. The prevalence of clinically significant symptoms of depression was higher than that observed in rheumatoid arthritis patients [46], elderly population without cognitive impairment [47], or French general population for whom the 12-month and lifetime prevalence rates were estimated to be 1.6% and 7.9% for dysthymia [48]. Recently, using the Composite International Diagnostic Interview (CIDI), Jewett *et al* found that the prevalence of 30-day, 12-month and lifetime major depressive disorder was 3.8%, 10.7% and 22.9% in SSc, respectively [49]. The high prevalence of clinically significant symptoms of depression in SSc may be related to numerous factors including body image, personality traits, and social network [16].

Surprisingly, only few studies have focused on clinically significant symptoms of anxiety in patients with SSc [12]. Using a dimensional scale, the Hamilton Anxiety Rating Scale, moderate and major anxiety was found in 64% and 19% of SSc patients, respectively [12], [50]. In addition, the prevalence of clinically significant symptoms of anxiety in patients with SSc appears higher than that observed in a French elderly population or a French general psychiatric outpatients sample, for whom the prevalence of current anxiety disorders was 14.2% [47] and 23% [51], respectively. However, as the HAD is a self-report questionnaire designed to only address levels of clinically significant symptoms of anxiety and depression and not to be used as a diagnostic tool, the rates of patients above the cut-off on the HAD cannot be compared to the rates of patients with confirmed diagnose based on structured interviews.

An important finding of our study is that most functional outcomes are significantly associated with clinically significant symptoms of depression and anxiety in SSc patients. As already observed [8], [9], [14], [16], overall disability as assessed by HAQ, was associated with clinically significant symptoms of depression and to a lesser extent of anxiety. Moreover in our study, patients exhibiting clinically significant symptoms of depression or of anxiety had greater hand disability, as assessed by the CHFS. Hand involvement is frequently encountered in patients with SSc and responsible for marked disability [52]. Even though hand mobility and capacity to perform activities of daily living seem to be maintained through the first years of SSc [53], hand involvement ultimately impacts global disability [28] and leads to socioeconomic burden [31]. Self-rated aesthetic impairment was also greater in patients with clinically significant symptoms of depression. One can assume that, as a consequence of skin involvement including sclerosis and telangiectasia, SSc patients may experience morphological changes prominently localized to the hands and face, and disfigurement [28] that may generate a distorted body image and contribute to the occurrence of psychological disturbances such as clinically significant symptoms of depression. Consistently, our result provided further evidence that body image distress is associated with depressive symptoms in SSc patients [54].

HRQoL PCS and MCS were strongly associated with both clinically significant symptoms of anxiety and depression. This finding is in agreement with previous reports. Using SF-36, Danieli *et al* found a strong correlation between altered HRQoL and the presence of clinically significant symptoms of depression in 76 Italian patients, whereas these symptoms poorly correlated with disease activity and severity indexes [46]. In a cross-sectional, multicenter study of 337 Canadian patients, Hudson *et al* confirmed the strong association of altered HRQoL assessed by the World Health Organization Disability Assessment Schedule II with clinically significant symptoms of depression [18]. It has been proposed that, in patients with SSc, poor HRQoL and symptoms of depression could be related to psychological distress symptoms, as well as to a number of personality traits, such as maladaptive defence and diminished sense of coherence leading to impaired psychological functioning [11].

Remarkably, we found no significant association of clinically significant psychiatric symptoms with disease-specific organ manifestations. This finding suggests that clinically significant symptoms of anxiety and depression in our SSc cohort could be rather related to perceived health status and disability, than to specific organ involvement or individual disease severity indicators. In contrast with some previous reports [18], our results do not support a strong relationship between medical symptoms and symptoms of depression. Specific symptoms including joint pain, gastrointestinal symptoms, respiratory symptoms have been previously reported as the best predictors of depression symptoms [18]. Other authors have also found that painful symptoms such as joint tenderness were associated with depression symptoms [8], [18], and that depression symptoms were more prevalent in SSc patients with pulmonary disease [12]. This discrepancy with our study might be explained by differences in disease severity or subsets, and recruitment type. Another plausible explanation could be the difference in data collection methods (i.e. self-reporting *vs* assessment by a physician). One cannot exclude that self-reporting might have overestimated functional symptoms, and underestimated specific symptoms. Likewise, some studies also suggest that SSc patients’ beliefs and emotional response are associated with the meaning they ascribe to their condition rather than its severity [55]. Among all clinical parameters evaluated, only general health status assessed by KPS score was found significantly decreased in patients with clinically significant symptoms of depression. Originally developed for cancer patients, because it strongly predicts cancer outcome [25], [56], the KPS score is also used in assessing acute or chronic conditions [57], [58]. As for cancer, our data support the use of the KPS score to predict outcome in SSc, because in addition to providing clinical estimates of patient’s physical state, performance, and prognosis, it is also associated with clinically significant symptoms of depression.

Our work has limitations including its cross-sectional design and the use of a self-report questionnaire for clinical features. Furthermore, our study did not assess association with social or economic status and was not designed to explore the cause for the observed differences, since the concurrent assessment of both outcome and associated variables did not allow for the evaluation of pathways of influence. Another limitation was the procedure used to recruit patients. Since patients belonged to the French patients association or were hospitalized in tertiary care units, they may not be representative of the whole French SSc population. Of note, the prevalence of clinically significant symptoms of depression and anxiety did not differ according to the type of recruitment. Patients had longstanding disease, which could imply more symptoms and disability. Indeed, HAQ scores were high but remained comparable to those reported from previous studies conducted in tertiary care settings [59]. Moreover, patients recruited from the patient association may have had more severe SSc than hospitalized patients [27]. However, in our sample, at the bivariate level, the two subgroups were comparable for all the outcomes assessed except for the frequence of telangiactasia and myalgia, and for the disease duration and MACTAR global scores (data not shown). In addition, even though the HAD questionnaire is an easy and cost-effective instrument to use routinely, self-rating psychiatric symptoms by the patients may lack sensitivity and specificity [45]. Recently published articles also raised some concerns about the ability of HAD scale to consistently differentiate between the constructs of anxiety and depression [60] and pointed out that the dimensionality of the HAD scale items is likely to be impacted by methodological artifacts [61], suggesting to abandon this scale or at least to revise it. Therefore, further prospective studies conducted in other cohorts of SSc patients using structured clinical interview performed by trained psychiatrists may be necessary to confirm our results. Finally, backward stepwise regression, though it is a commonly used and published modelling method, may have some limitations with main concerns regarding overfitting. Indeed, it has been previously reported that predictors detected using this method, could actually not be related to the response in the population in some cases, and could be pure noise [62].

In summary, we confirm the high prevalence of clinically significant anxious and depressive symptoms among patients with SSc and provide evidence for their association with increased disability and poorer HRQoL, rather than with SSc-specific clinical manifestations. Our results suggest that special attention should be paid to detect clinically significant symptoms of anxiety and/or depression in patients with SSc in order to propose suitable interventions. Indeed, when clinically significant psychiatric symptoms accompanies a medical illness, each one worsens the other [63]. Consistently, people who receive treatment for co-occurring clinically significant psychiatric symptoms often show improvement in their overall medical condition and better quality of life, and comply better with general medical care [64]. However, randomized controlled trials are required to further examine the benefit of adequate screening and treatment of mood and anxiety disorders in SSc.
